# Supplementary material for: Repurposing α-Adrenoreceptor Blockers as Promising Anti-Virulence Agents in Gram-Negative Bacteria
Source: Antibiotics (Basel). 2022 Jan 29;11(2):178. doi: 10.3390/antibiotics11020178 (PMC8868568; doi:10.3390/antibiotics11020178)
Supplement: Supplementary file 1 [file antibiotics-11-00178-s001.zip › antibiotics-1539664-supplementary.pdf]

Repurposing of α-adrenergic blockers as promising anti-virulence agents in Gram-negative bacteria

Ahmad J. Almalki [1,*], Tarek S. Ibrahim [1], Sameh S. Elhady [2], Khaled M. Darwish [3] and Wael A. H. Hegazy [4,*]

[1] Department of Pharmaceutical Chemistry, Faculty of Pharmacy, King Abdulaziz University, Jeddah 21589, Saudi Arabia; tmabrahem@kau.edu.sa
[2] Department of Natural Products, Faculty of Pharmacy, King Abdulaziz University, Jeddah 21589, Saudi Arabia; ssahmed@kau.edu.sa
[3] Department of Medicinal Chemistry, Faculty of Pharmacy, Suez Canal University, Ismailia 41522, Egypt; khaled_darwish@pharm.suez.edu.eg
[4] Department of Microbiology and Immunology, Faculty of Pharmacy, Zagazig University, Zagazig 44519, Egypt
* Correspondence: ajalmalki@kau.edu.sa (A.J.A.); waelmhegazy@daad-alumni.de (W.A.H.H.); Tel.: +20-110-118-8800 (W.A.H.H.)

Additional Experimental Details

1) **Figure S1.** Superimposing the co-crystallized (magenta sticks) and redocked (yellow sticks) ligands.
2) **Figure S2.** 3D-representation of the binding site topology at the two bacterial LuxR-type quorum-sensing transcription factors.
3) **Figure S3.** The architecture of two bacterial LuxR-type quorum sensing transcription factors.
4) **Table S1.** Descriptive ligand-QscR *P. aeruginosa* binding interactions through directed flexible docking protocol.
5) **Table S2.** Descriptive ligand-CviR *C. violaceum* binding interactions through directed flexible docking protocol.
6) **Table S3.** Estimated ΔRMSF values for ligand-QscR *P. aeruginosa* proteins along the whole MD simulation.
7) **Table S4.** Estimated ΔRMSF values for ligand-CviR *C. violaceum* proteins along the whole MD simulation.
8) **Table S5.** List of top putative active sites obtained from the MOE-Alpha Site Finder module.
9) **Table S6.** List of lining residues comprising the identified canonical binding site of the investigated QS proteins.
10) **Table S7.** Sequences of the used primers.

(A) (B)

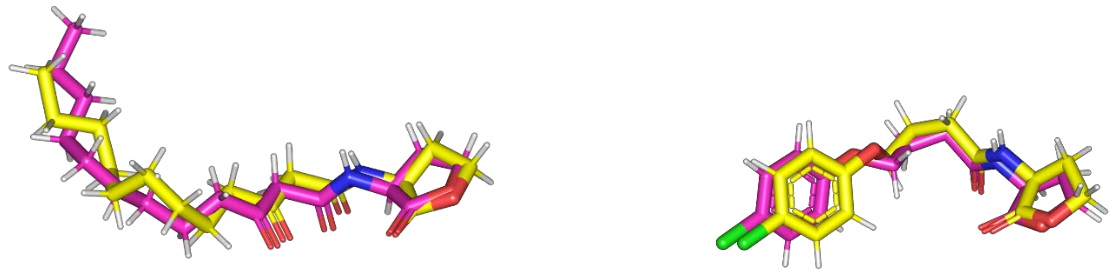

Figure S1. Superimposing the co-crystallized (magenta sticks) and redocked (yellow sticks) ligands. (**A**) QscR *P. aeruginosa*; (**B**) CviR *C. violaceum* for validating the adopted directed docking protocol.

(A) (B)

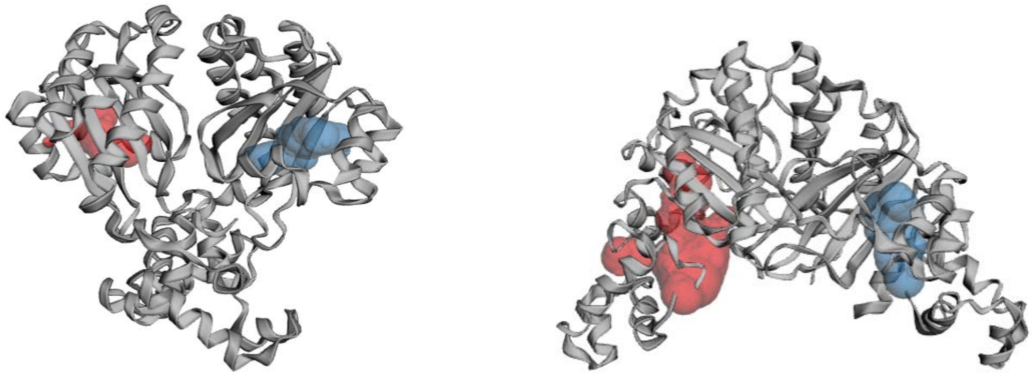

Figure S2. 3D-representation of the binding site topology at the two bacterial LuxR-type quorum-sensing transcription factors. (**A**) QscR *P. aeruginosa*; (**B**) CviR *C. violaceum*. Putative pockets were calculated via the online Computed Atlas of Surface Topography of proteins (CASTp; http://sts.bioe.uic.edu/castp/index.html) using 1.4 Å radius probe, visualized as surface 3D-representation, and colored in different colors (blue and red) for each target protomer. Estimated pocket area and volume were analytically calculated using the Richard's solvent-accessible surface model.

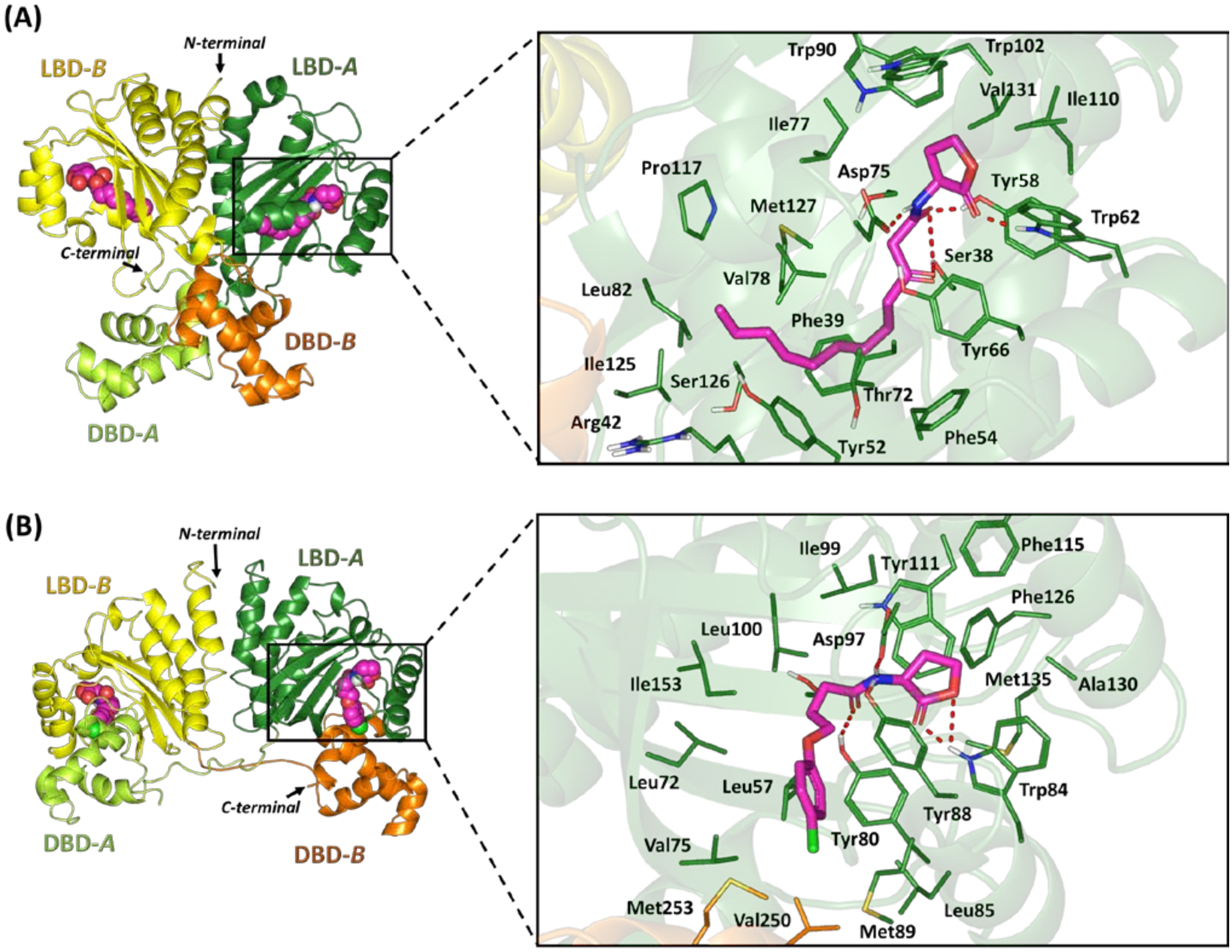

Figure S3. The architecture of two bacterial LuxR-type quorum sensing transcription factors. Left panels are overall cartoon representation of the (**A**) QscR from *P. aeruginosa* PDB entry: 3SZT, (**B**) CviR from *C. violaceum* PDB entry: 3QP5, where each protomer is colored differently in regard to its ligand-binding domain (LBD) and DNA-binding domain (DBD) as light/dark green and dark/light orange for protomer-A and -B, respectively. Ligands are represented as magenta spheres. Right panels are the overlay of each co-crystallized ligand (magenta sticks) at the binding site of its respective bacterial LuxR-type quorum sensing transcription factor; (**A**) O-C12-HSL, and (**B**) HLC. Polar interactions, represented as hydrogen bonds, are illustrated as red dashed-lines and only residues (green lines), located within 4 Å radius of bound ligand, are displayed and labeled with sequence number. Crystallized water molecule bridging the ligand/residue interactions is shown as red sphere.

Table S1. Descriptive ligand-QscR *P. aeruginosa* binding interactions through directed flexible docking protocol

Compound	Ligand-target interaction description<br>[Type; Length (Å); Angle (°); Binding Residues]
**Terazosin**	H-bond; 2.4 **Å**; 143 °; Ser38 (sidechain O**H** with piperazine-**N**)<br>H-bond; 3.0 **Å**; 129 °; Trp62 (sidechain N**H** with furan **O**)<br>H-bond; 3.3 **Å**; 127 °; Tyr66 (sidechain O**H** with quinazoline **N**³)<br>H-bond; 2.7 **Å**; 124 °; Met127 (mainchain N**H** with 7-**O**CH_3)<br>H-bond; 3.3 **Å**; 123 °; Ser129 (sidechain O**H** with quinazoline **N**¹)<br>π-H interaction; 2.9 **Å**; Phe54<br>π-H interaction; 3.7 **Å**; Trp102<br>van der Waals; 3.9 **Å**; Arg42 sidechain C*β*
**Prazosin**	H-bond; 2.7 **Å**; 131 °; Ser38 (sidechain **O**H with quinazoline NH**H**)<br>H-bond; 3.0 **Å**; 174 °; Tyr58 (sidechain O**H** with piperazine **N**)<br>H-bond; 2.0 **Å**; 134 °; Trp62 (sidechain O**H** with C=**O**)<br>H-bond; 1.6 **Å**; 125 °; Met127 (mainchain N**H** with quinazoline NH**H**)<br>H-bond; 2.6 **Å**; 125 °; Ser129 (sidechain O**H** with quinazoline **N**³)<br>π-π interaction; 4.3 **Å**; Phe54<br>π-H interaction; 2.7 **Å**; Trp102<br>van der Waals; 4.0 **Å**; Arg42 sidechain C*β*
**HLC**	H-bond; 2.8 **Å**; 128 °; Ser38 (sidechain O**H** with amide C=**O**)<br>H-bond; 2.1 **Å**; 148 °; Tyr58 (sidechain N**H** with aryloxy-**O**)<br>H-bond; 2.0 **Å**; 147 °; Trp62 (sidechain N**H** with lactone C=**O**)<br>H-bond; 2.6 **Å**; 149 °; Tyr66 (sidechain O**H** with amide C=**O**)<br>H-bond; 2.4 **Å**; 129 °; Asp75 (sidechain O**H** with amide C=**O**)<br>π-π interaction; 4.5 **Å**; Phe54<br>π-H interaction; 2.7 **Å**; Trp90<br>van der Waal; 4.6 **Å**; Arg42 sidechain C*β*

Table S2. Descriptive ligand-CviR *C. violaceum* binding interactions through directed flexible docking protocol

Compound	Ligand-target interaction description<br>[Type; Length (Å); Angle (°); Binding Residues]
**Tamsulosin**	H-bond; 3.1 **Å**; 123 °; Asp97 (sidechain C=**O** with sulphonamide N**H**)<br>H-bond; 2.4 **Å**; 137 °; Tyr80 (sidechain O**H** with sulphonamide S=**O**)<br>H-bond; 3.3 **Å**; 121 °; Ser155 (sidechain O**H** with sulphonamide S=**O**)<br>π-π interaction; 4.9 **Å**; Tyr80<br>π-H interaction; 3.8 **Å**; Tyr88<br>van der Waals; 4.0 **Å**; Asn92 sidechain C*β*
**Terazosin**	H-bond; 3.1 **Å**; 128 °; Tyr80 (sidechain O**H** with piperazine **N**)<br>H-bond; 3.3 **Å**; 128 °; Met89 (mainchain N**H** with quinazoline-7-**O**H)<br>H-bond; 2.1 **Å**; 125 °; Trp84 (sidechain O**H** with tetrahydrofuranoyl C=**O**)<br>H-bond; 3.2 **Å**; 128 °; Trp111 (sidechain O**H** with tetrahydrofuran **O**)<br>π-H interaction; 3.0 **Å**; Phe54<br>π-H interaction; 2.7 **Å**; Trp111<br>van der Waals; 4.0 **Å**; Arg42 sidechain C*β*
**Prazosin**	H-bond; 3.3 **Å**; 127 °; Leu72 (mainchain N**H** with quinazoline-6-**O**H)<br>H-bond; 2.1 **Å**; 130 °; Trp84 (sidechain N**H** with furanoyl C=**O**)<br>H-bond; 3.3 **Å**; 137 °; Tyr88 (mainchain N**H** with quinazoline-NH_2)<br>π-H interaction; 3.7 **Å**; Leu72<br>π-H interaction; 3.6 **Å**; Tyr80<br>π-π interaction; 3.1 **Å**; Tyr88<br>π-π interaction; 4,2 **Å**; Trp111
**HLC**	H-bond; 2.5 **Å**; 158 °; Tyr80 (sidechain O**H** with amide C=**O**)<br>H-bond; 2.0 **Å**; 169 °; Trp84 (sidechain N**H** with lactone C=**O**)<br>H-bond; 2.8 **Å**; 121 °; Trp84 (sidechain N**H** with lactone **O**)<br>H-bond; 2.3 **Å**; 161 °; Asp97 (sidechain C=**O** with amide N**H**)<br>π-H interaction; 4.0 **Å**; Tyr80<br>π-π interaction; 5.1 **Å**; Tyr88<br>π-H interaction; 2.8 **Å**; Trp111

Table S3. Estimated ΔRMSF[a] values for ligand-QscR *P. aeruginosa* proteins along the whole MD simulation.

Pocket Residues	HLC	Comp.5	Comp.6
**Ser38**	0.18	**0.41**	**0.39**
**Phe39**	0.02	0.28	**0.30**
**Gly40**	-0.05	0.16	0.20
**Ala41**	0.14	**0.35**	0.27
**Arg42**	0.02	**0.36**	0.29
**Tyr52**	-0.64	-0.03	-0.09
**His53**	-0.25	-0.18	-0.14
**Phe54**	-0.44	-0.20	-0.10
**Ser56**	-0.51	-0.07	-0.05
**Tyr58**	-0.10	0.22	0.11
**Trp62**	-0.08	**0.56**	**0.40**
**Lys63**	-0.40	0.17	0.04
**Tyr66**	-0.77	0.06	-0.13
**Ile67**	-1.19	-0.21	-0.39
**Thr72**	-0.88	-0.67	-0.74
**Asp75**	**0.31**	0.29	0.28
**Ile77**	**0.30**	**0.43**	0.10
**Val78**	**0.37**	**0.49**	**0.30**
**Leu82**	**0.36**	**0.56**	0.26
**Trp90**	**0.34**	**0.54**	**0.44**
**Phe101**	-0.49	0.17	-0.11
**Trp102**	-0.11	**0.51**	0.21
**Ala105**	-0.58	-0.07	-0.28
**Ile110**	-0.05	**0.38**	0.20
**Ile125**	**0.74**	**0.93**	**0.84**
**Met127**	**0.82**	**0.94**	**0.90**
**Ser129**	**0.91**	**1.03**	**1.00**

[a] Relative difference root-mean-square fluctuation (ΔRMSF) was estimated for each ligand-associated QscR protein relative to the apo/unliganded state. Residues showing significant immobility are with ΔRMSF > 0.30 Å cut-off are in bold red color and highlighted.

Table S4. Estimated ΔRMSF[a] values for ligand-CviR *C. violaceum* proteins along the whole MD simulation.

Pocket Residues	HLC	Comp.4	Comp.5	Comp.6
**Leu57**	**0.80**	**0.61**	**0.47**	**0.57**
**Ile69**	-0.72	-0.69	-0.39	-0.34
**Gln70**	-0.87	-0.85	-0.74	-0.42
**Arg71**	-0.70	-0.71	-0.75	-0.25
**Leu72**	0.18	-0.62	-0.62	-0.31
**Val75**	-0.14	-1.22	-0.52	-0.67
**Asn77**	-0.72	-0.73	0.19	-0.09
**Tyr80**	**0.49**	**0.32**	**0.88**	**0.57**
**Trp84**	0.26	0.21	**0.44**	0.29
**Leu85**	**0.31**	0.02	**0.62**	0.19
**Tyr88**	0.10	-0.72	0.28	-0.08
**Met89**	**0.52**	**0.32**	**0.71**	**0.40**
**Ala94**	0.23	**0.35**	**0.34**	**0.34**
**Gln95**	0.25	**0.58**	**0.40**	**0.33**
**Asp97**	**0.43**	**0.38**	0.25	0.28
**Pro98**	-0.37	-0.67	-0.24	-0.46
**Ile99**	**0.44**	**0.58**	**0.53**	**0.56**
**Leu100**	**0.39**	**0.98**	**0.67**	**0.67**
**Arg101**	-0.67	-0.94	-0.42	-0.59
**Trp111**	**1.25**	**0.76**	**0.35**	**0.95**
**Phe115**	0.21	**0.36**	**0.61**	**0.65**
**Phe126**	-0.70	-1.31	-0.27	-0.49
**Ala130**	-0.55	-1.09	-0.07	-0.35
**Met135**	-0.16	-0.58	0.37	-0.03
**Thr140**	-0.81	-1.10	-0.30	-0.70
**Ile153**	**1.38**	**1.06**	**0.88**	**1.11**
**Ser155**	**0.84**	**0.64**	**0.40**	**0.60**
**Val250**	**1.31**	**2.17**	**0.33**	**1.71**

[a] Relative difference root-mean-square fluctuation (ΔRMSF) was estimated for each ligand-associated CviR protein relative to the apo/unliganded state. Residues showing significant immobility are with ΔRMSF > 0.30 Å cut-off are in bold red color and highlighted.

Table S5. List of top putative active sites obtained from the MOE-Alpha Site Finder module

	Site №.	Size [a]	PLB [b]	Hyd [c]	Side [d]
**QscR**	**1**	**126**	**3.68**	**52**	**90**
	**2**	**83**	**2.56**	**46**	**66**
	**3**	**62**	**1.38**	**28**	**56**
**CviR**	**1**	**93**	**3.06**	**42**	**61**
	**2**	**115**	**2.96**	**52**	**73**
	**3**	**100**	**2.17**	**22**	**48**

[a] Size column indicates the number of Alpha Spheres comprising the site, which are a generalization of convex hulls. These spheres contact four atoms and do not contain any atom. [b] PLB column indicates the Propensity for Ligand Binding score depending on the composition of the contact residues within the target pocket. [c] Hyd column indicates the number of hydrophobic contact atoms in the binding site. [d] Side column indicates the number of sidechain contact atoms in the binding site. The list is sorted by the PLB column (descending order). Selection of each target's canonical binding site was done based on the MOE site scoring function indicating the propensity for ligand binding score depending on the composition of the contact residues within the target pocket. However, site refinement was then proceeded through matching the MOE-obtained site with that obtained from the CASTp on-line server, the inclusion of the crystalline ligands, as well as crucial residues reported within the current literature. The selected canonical binding site for each target is highlighted.

Table S6. List of lining residues comprising the identified canonical binding site of the investigated QS proteins

QS Target	Lining residues of the identified active pocket
**QscR *P. aeruginosa***	Ser38, Phe39, Gly40, Ala41, Arg42, Tyr52, His53, Phe54, Ser56, Tyr58, Trp62, Lys63, Tyr66, Ile67, Thr72, Asp75, Ile77, Val78, Leu82, Trp90, Phe101, Trp102, Ala105, Ile110, Ile125, Met127, Ser129
**CviR *C. violaceum***	Leu57, Ile69, Gln70, Arg71, Leu72, Val75, Asn77, Tyr80, Trp84, Leu85, Tyr88, Met89, Ala94, Gln95, Asp97, Pro98, Ile99, Leu100, Arg101, Trp111, Phe115, Phe126, Ala130, Met135, Thr140, Ile153, Ser155, Val250

Table S7. Sequences of the used primers

Target gene	Sequence (5′–3′)	Reference
lasI	**For:** CTACAGCCTGCAGAACGACA<br>**Rev:** ATCTGGGTCTTGGCATTGAG	[3]
lasR	**For:** ACGCTCAAGTGGAAAATTGG<br>**Rev:** GTAGATGGACGGTTCCCAGA	[3]
rhlI	**For:** CTCTCTGAATCGCTGGAAGG<br>**Rev:** GACGTCCTTGAGCAGGTAGG	[3,49]
rhlR	**For:** AGGAATGACGGAGGCTTTTT<br>**Rev:** CCCGTAGTTCTGCATCTGGT	[3]
pqsA	**For:** TTCTGTTCCGCCTCGATTTC<br>**Rev:** AGTCGTTCAACGCCAGCAC	[3]
pqsR	**For:** AACCTGGAAATCGACCTGTG<br>**Rev:** TGAAATCGTCGAGCAGTACG	[3]
pmrA	**For:** CACCAGGTGACCCTGTCC<br>**Rev:** CGTAGAGGCTCTGCTCCAGT	[107]
pmrB	**For:** CCTGAAAACCGCCTACCGGA<br>**Rev:** TTCGGTGGCAAGGTCGAGCA	[108]
rpoD	**For:** GGGCGAAGAAGGAAATGGTC<br>**Rev:** CAGGTGGCGTAGGTGGAGAAC	[3]
